# Differential Response to Non‐Surgical Periodontal Therapy Between Intrabony and Suprabony Defects: A Retrospective Analysis

**DOI:** 10.1111/jcpe.14181

**Published:** 2025-05-19

**Authors:** Alessandro Cuozzo, Mishika Arora, Lorenzo Marini, Joshua Hurley, Zainab Malaki, Jing Kang, Luca Ramaglia, Luigi Nibali

**Affiliations:** ^1^ Periodontology Unit Centre for Host Microbiome Interactions, Faculty of Dentistry, Oral and Craniofacial Sciences, King's College London London UK; ^2^ Periodontology Unit, Department of Neuroscience, Reproductive and Odontostomatological Sciences University of Naples Federico II Naples Italy; ^3^ Section of Periodontics, Department of Oral and Maxillofacial Sciences Sapienza University of Rome Rome Italy

**Keywords:** bone loss, intrabony, non‐surgical therapy, osseous defects, periodontitis, suprabony

## Abstract

**Aim:**

To assess the clinical response to Non‐Surgical Periodontal Therapy (NSPT) between suprabony and intrabony defects.

**Materials and Methods:**

Totally 200 NSPT patients' records from the King's College London—Oral, Dental and Craniofacial Biobank were included. Periapical radiographs of sites with Periodontal Probing Depth (PPD) > 4 mm were assessed. Changes in periodontal measurements between baseline and three‐month re‐evaluation were examined and compared across suprabony and intrabony sites. Multilevel analysis was carried out to assess the relative contribution of site‐, tooth‐, patient‐ and treatment‐related factors on the clinical outcomes.

**Results:**

Although intrabony defects showed higher PPD reduction after NSPT compared with suprabony defects, this was a function of initial deeper PPD. In fact, suprabony defects outperformed intrabony defects for ‘pocket closure’ at every initial PPD threshold and overall were 2.60 times more likely to achieve ‘pocket closure’. Defect morphology was one of several factors affecting treatment outcomes. The relative contribution to ‘pocket closure’ was 64.9% for site‐, 26.1% for patient‐, 9.0% for tooth‐ and 1% for treatment‐related factors.

**Conclusion:**

Bone defect morphology influences clinical outcome of NSPT, with suprabony defects being twice as likely to achieve pocket closure and resulting in 0.6 mm more PPD reduction compared with intrabony defects.

## 1 Introduction

Periodontal bony defects are usually sub‐classified into rarer ‘intrabony (vertical/angular) defects’, where the base of the pocket is apical to the residual bone crest, and more common ‘suprabony (horizontal) defects’, where the base of the pocket is at the level of the residual bone crest (Papapanou and Tonetti 2000).

One of the main outcomes of Non‐Surgical Periodontal Therapy (NSPT) is the ‘pocket closure’, defined as achieving a Pocket Probing Depth (PPD) ≤ 4 mm (Tomasi et al. 2007). While this goal could be more easily achievable for suprabony defects, intrabony defects are thought to respond less favourably to NSPT. In the case of osseous defects with at least 3 mm of intrabony component, the recent guidelines of the European Federation of Periodontology (EFP) strongly recommend a regenerative surgical approach if PPDs > 5 mm with Bleeding on Probing (BoP) persist following steps 1 and 2 of periodontal treatment (Sanz et al. 2020).

However, recent evidence suggests that NSPT can lead to significant improvements in intrabony defects, both clinically and radiographically (Nibali et al. 2018; Anoixiadou et al. 2022). Furthermore, a recent systematic review from our group showed that there is limited evidence to suggest that defect morphology affects the clinical response to NSPT (Marini et al. 2024). Most of the evidence relates to the non‐surgical response of suprabony defects, while few studies involving intrabony defects or performing the comparison based on the defect morphology are reported.

Therefore, the aim of this study was to evaluate whether intrabony defects have a differential response to NSPT compared to suprabony defects. The null hypothesis was that intrabony defects had the same prevalence of ‘pocket closure’ at 3 months after NSPT as suprabony defects. A secondary aim was to explore the relative contribution of site‐, tooth‐, patient‐ and treatment‐related factors to the healing of periodontal pockets after NSPT.

## 2 Materials and Methods

### 2.1 Study Design

This was a cross‐sectional study on samples collected from patients taking part in the King's College London‐Oral, Dental and Craniofacial Biobank. The Biobank was granted ethics approval by the East of England—Cambridge East Research Ethics Committee (reference number EE/0241). Each patient gave written consent to take part in the Biobank recruitment which occurred between December 2020 and March 2023 in Periodontology new patients' clinic in Guy's & St Thomas Foundation Trust (GSTFT) hospital. Specific approval for the release of data and samples for this study was granted by the Biobank Management Committee (Biobank reference 016). Among 723 patients who had consented to the Biobank up to March 2023, a total of 200 consecutive patients who had received NSPT were included in the analysis.

### 2.2 Clinical Examination

Following consent, demographic parameters were collected, along with dental and medical history. Diabetes and self‐reported smoking habit (number of cigarettes/days, years of smoking, former smokers) were also recorded. ‘Uncontrolled diabetes’ was defined as HbA1c > 6.5% at pre‐treatment chair‐side testing. Height, weight and waist measurements were taken at the study visit. Tooth type (molar or non‐molar) was reported, and the following periodontal measurements were taken at six sites/tooth by Biobank examiners using a UNC‐15 periodontal probe: dichotomous Full‐Mouth Plaque Score (FMPS) (Guerrero et al. 2005), Probing Pocket Depth (PPD), Recession (REC), Bleeding on Probing (BoP) (Ainamo and Bay 1975), tooth mobility (Laster et al. 1975) and furcation involvement (Hamp et al. 1975). Clinical Attachment Level (CAL) was calculated as PPD + REC. Periodontal diagnosis was based on the current classification of periodontal diseases (Caton et al. 2018). All sites affected by periodontitis and with PPDs > 4 mm were then selected in a dedicated database.

### 2.3 Radiographic Analysis

Available digital long cone periapical radiographs of all sites with PPDs > 4 mm at the first study visit were sought and transferred into a dedicated software measurement system (Emago, Oral Diagnostic Systems, Amsterdam, Netherlands).

**FIGURE 1:**
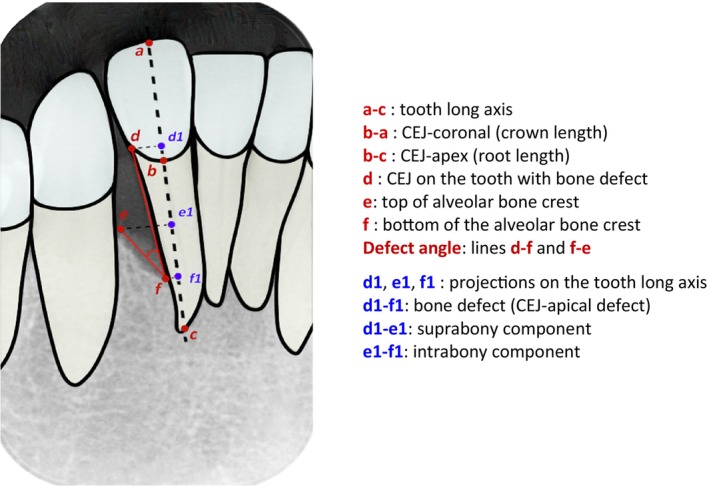
Schematic representation of reference points for radiographic bone loss analysis.

A calibrated examiner (author A.C.) assessed radiographs at each mesial and distal site and measured radiographic bone loss using specific landmarks (Figure 1) based on a previous study (Nibali et al. 2011).

Intra‐class Correlation Coefficients (ICC) were obtained on 7 different parameters (Supporting Information 1) and ranged between 0.811 (95% CI: 0.408–0.949) and 0.996 (95% CI: 0.984–0.999). Sites were classified based on the definitions below (Papapanou and Wennström 1991):

– Suprabony defect: base of defect at the level of the residual bone crest, with an apical‐coronal bone levels difference < 2 mm;
– Intrabony: base of defect apical to the residual bone crest, with an apical‐coronal bone level difference ≥ 2 mm;

Missing teeth, impacted third molars, semi‐impacted teeth, dental implants, deciduous teeth, residual roots, unclear or unavailable periapical radiographs were also recorded. Inter‐root defects (e.g., craters/ramps) were not included in the analysis due to the likelihood of misclassifying them as suprabony defects by radiographic investigation.

### 2.4 Non‐Surgical Periodontal Therapy (NSPT)

Step 1 of periodontal therapy was carried out in each patient, followed by subgingival debridement (step 2) in all sites exhibiting PPDs > 3 mm and when FMPS was < 40%. NSPT was performed by undergraduate and postgraduate students, hygienists, or staff members in the Periodontology Unit using ultrasonic instruments, hand instruments, a combination of both, or a MINST (Minimally Invasive Non‐Surgical Therapy) technique with ultrasonic devices. Students were supervised by periodontal specialists at all times during treatment.

Data related to NSPT were recorded, including initial and follow‐up FMPS (where possible), therapist, use and type of adjunct, number of NSPT visits, type of instrumentation used, any further periodontal/dental treatments (including surgical therapy), date of final NSPT and of first periodontal re‐evaluation, the final clinical outcomes and the final appointment date.

### 2.5 Periodontal Re‐Assessment

Patients were reassessed approximately 3 months after completion of NSPT. Changes in periodontal measurements between baseline and re‐evaluation time were collected for all bony defects. ‘Pocket closure’ was defined as PPD reducing from > 4 mm to < 5 mm with no BoP (BoP‐) following treatment.

### 2.6 Statistical Analysis and Sample Size Calculation

‘Pocket closure’ is reported to occur in 74% of osseous defects (including intrabony and suprabony) following NSPT (Suvan et al. 2020). The literature reports that suprabony defects are approximately 10 times more common than intrabony defects (Papapanou et al. 1988; Persson et al. 1998; Müller and Ulbrich 2005; Jayakumar et al. 2010). Based on the hypothesis that ‘pocket closure’ occurs in 80% of suprabony defects (more common) and 50% of intrabony defects (less common), 60 intrabony defects and 600 suprabony defects would give 90% power to observe such difference if sites were considered as independent samples. By assuming the intra‐class correlations (ICC) between sites of the same tooth and teeth from the same patients are 0.5 and 0.2, respectively, and that each tooth has 2 sites recorded and each patient has 8 teeth recorded for defect, the minimum required number of patients is 150 (design effect of site = 1 + ICC_site × (N_sitePerTooth − 1) = 1 + 0.5 × (2 − 1) = 1.5; N_teethNeeded = N_sites × Design effect of site/N_sitePerTooth = 660 × 1.5/2 = 495; Design effect of teeth = 1 + ICC_tooth × (N_toothPerPatient − 1) = 1 + 0.2 × (8 − 1) = 2.4; N_patients = N_teethNeeded × Design effect of teeth/N_toothPerPatient = 495 × 2.4/8 = 150). Compensating for a potentially higher ICC of tooth and site, a choice was made to include the first 200 consecutive patients with the required data available. Data from all included patients were entered into a spreadsheet and proofed for entry errors.

Descriptive statistics were performed for patients' demographic (age, BMI, gender, ethnicity, smoking status and diabetes) and clinical characteristics (brushing frequency, daily interdental cleaning, periodontal measurements, defect definition, defect depth, suprabony and intrabony components). Continuous variables were presented as mean with standard deviation, while categorical variables as frequency with percentage.

Periodontal measurements included average BoP, PPD and CAL at baseline and at re‐assessment. The associations between the clinical outcomes (pocket closure, PPD reduction, CAL gain) and the baseline measures with the exposure variable (‘intrabony versus suprabony’) were assessed with a multilevel modelling (MLM), including a sub‐group ‘without adjuncts’ analysis and with further adjustment of the clustering effect among sites of the same teeth, and teeth of the same patients. Confounders were also adjusted for the MLM. Changes of *R* ^2^ were calculated to assess the relative contribution of treatment variables to the model.

The proportion of variance explained at site, tooth and patient level was calculated as their relative contribution to the clinical responses.

A sub‐group analysis was further conducted using MLM within only samples of intrabony defects on the outcome ‘pocket closure’, adjusted by initial PPD, age, gender and diabetes. Data analysis was performed using SPSS for Windows version 27.0 (IBM Corp., Armonk, NY) and Rstudio version 2024.04.2 with various packages. Significance level was set at 0.05.

## 3 Results

### 3.1 Patient Population

Table 1 reports demographic and clinical characteristics of all included patients. Most patients were male (58%) and Caucasian (45%), with a mean BMI of 26.5 ± 5 kg/m^2^ and a mean age of 47 ± 13 years. Uncontrolled diabetes (HbA1c ≥ 6.5%) was reported in 5.5% of cases, while more than half of the sample (57%) were never‐smoking patients.

**TABLE 1:** Demographic and clinical characteristics of patients included in the study.

Age (years) (mean ± SD)		47 ± 13
BMI (kg/m^2^) (mean ± SD)		26.5 ± 5
Gender	Male (*n*)	116; 58%
	Female (*n*)	84; 42%
Ethnicity	Caucasian (*n*)	90; 45%
	Afro‐Caribbean (*n*)	51; 25.5%
	Other/mixed (*n*)	59; 29.5%
Smoking	Never (*n*)	114; 57%
	Former (*n*)	69; 34.5%
	Current (*n*)	17; 8.5%
Diabetes type II	Yes (*n*)	21; 10.5%
	Uncontrolled (HbA1c ≥ 6.5%)	11; 5.5%
	Controlled (HbA1c < 6.5%)	10; 5%
	No (*n*)	179; 89.5%
Brushing frequency	< 2/day (*n*)	18; 9%
	At least 2/day (*n*)	182; 91%
Daily interdental cleaning	Yes (*n*)	174; 87%
	No (*n*)	16; 13%

Almost all patients (91%) reported a brushing frequency of at least twice a day, while the daily use of interdental hygiene devices was reported in 87% of cases. Periodontitis staging and grading are reported in Supporting Information 2.

### 3.2 Clinical and Radiographic Characteristics of Examined Sites

A total of 11,160 interproximal sites across 5587 teeth from 200 patients at baseline were included. Table 2 shows the periodontal and radiographic characteristics of all examined sites at baseline. Out of the included sites (excluding impacted third molars, semi‐impacted teeth, dental implants, sites adjacent to dental implants, deciduous teeth and residual roots), 30.2% had a PPD ≥ 5 mm, while 18.6% and 10.9% had a PPD ≥ 6 mm and ≥ 7 mm, respectively. Average PPD and CAL were 3.77 and 4.28 mm in buccal and lingual interproximal sites combined, while the average BoP was 39.54%. Baseline FMPS was 38% ± 22.8%, although it could only be retrieved for 50 cases.

**TABLE 2:** Periodontal and radiographic characteristics of examined sites at baseline with available periapical radiographs.

Periodontal measurements	Average BoP (%) (mean ± SD)	39.54 ± 26.30
	Average CAL (mm) (mean ± SD)	4.28 ± 2.41
	Average PPD (mm) (mean ± SD)	3.77 ± 2.07
	Number of sites with PPD ≥ 5 mm (*n*)	6093; 30.20%
	Number of sites with PPD ≥ 6 mm (*n*)	3755; 18.61%
	Number of sites with PPD ≥ 7 mm (*n*)	2203; 10.90%
Defect definition	No bone loss (*n*)	1050; 17.31%
	Suprabony (*n*)	4258; 70.21%
	Intrabony (*n*)	454; 7.48%
	Others (impacted/semi‐impacted teeth, deciduous teeth, residual roots) (*n*)	37; 0.61%
	Unclear (*n*)	266; 4.39%
Overall defect depth (mm) (mean ± SD)		3.44 ± 2.34
Average suprabony component (mm) (mean ± SD)		3.34 ± 1.97
Average intrabony component (mm) (mean ± SD)		0.39 ± 1.10

Radiographic characteristics of 22,320 study sites with available periapical radiographs were recorded and included in the analysis. Of those which could be clearly detected and classified radiographically, a total of 4258 bony defects (70.21%) were defined as ‘suprabony’, while 454 (7.48%) were classified as ‘intrabony’. The overall defect depth was on average 3.44 mm, with an average suprabony component of 3.34 mm and of 0.39 mm for the intrabony component.

### 3.3 Treatment Response

A total of 60 teeth were extracted following baseline. NSPT was carried out by 84 different clinicians, with an average of approximately two NSPT sessions per patient (1.83 ± 0.96). Based on available data, most clinicians adopted a combination of ultrasonic and hand instruments, while 30 patients (15.3%) were treated with adjuncts (Supporting Information 3). Among these, amoxicillin (alone/in combination with metronidazole) and azithromycin were used in 21 (70% of cases with adjuncts) and in 6 (20%) cases, respectively. Further data (including dosage) are reported in Supporting Information 3.

Patients were reassessed on average 102.97 ± 73.64 days after treatment. At re‐evaluation, 54.1% of sites (intrabony and suprabony) with PPD > 4 mm at baseline (*n* = 6093) converted to PPD sites < 5 mm and BoP‐ (‘pocket closure’; *n* = 3294).

The average PPD reduction in all sites was 0.58 mm and the average CAL gain was 0.26 mm. Bleeding on Probing (BoP) decreased from 39.54% at baseline to 21%, while FMPS of patients with also recorded baseline FMPS decreased from 38% ± 20.6% to 21.8% ± 17.5%. FMPS of patients with only follow‐up FMPS (baseline FMPS not available) (*n* = 162) was 21.0% ± 19.3%.

The breakdown of PPD reduction and CAL gain by initial PPD is reported in Table 3. Sites with baseline PPD ≥ 7 mm and between 4 and 6 mm showed an average PPD reduction of 2.69 mm and 1.18 mm, respectively, while sites with initial PPD between 1 and 3 mm presented a slight PPD increase (−0.16 ± 1.09 mm), as well as CAL gain (−0.32 ± 1.42 mm). Conversely, higher CAL gain was observed for sites with a baseline PPD ≥ 7 mm (1.80 mm) and between 4 and 6 mm (0.76 mm).

**TABLE 3:** Breakdown of PPD reduction and CAL gain by initial pocket depth.

Initial PPD	PPD reduction (mm) (mean ± SD)	CAL gain (mm) (mean ± SD)
1‐3 mm (*n* = 10,669)	−0.16 ± 1.09	−0.32 ± 1.42
4‐6 mm buccal (*n* = 6185)	1.18 ± 1.50	0.76 ± 1.85
≥ 7 mm buccal (*n* = 2001)	2.69 ± 2.17	1.80 ± 2.37

### 3.4 Association Between Treatment Response and Radiographic Defect Morphology

**FIGURE 2:**
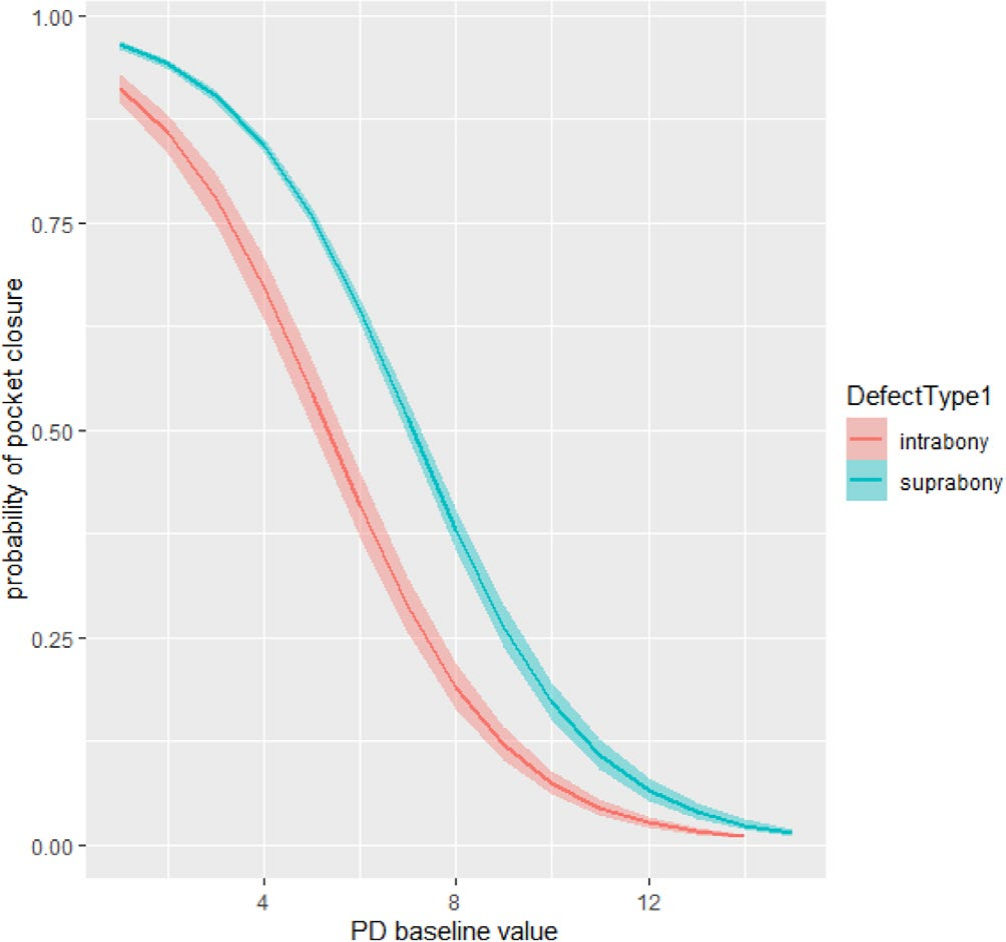
Proportion of ‘pocket closure’ at re‐evaluation according to initial PPD from 5 to 10 mm divided by suprabony and intrabony defects. All comparisons between suprabony/intrabony are *p* < 0.001, except 8 mm (*p* = 0.002), 9 mm (*p* = 0.171) and 10 mm (*p* = 0.141).

Table 4 reports periodontal clinical data at baseline and after NSPT according to radiographic defect type. Intrabony defects had higher mean PPD reduction at re‐evaluation (0.91 mm) compared with suprabony defects (0.72 mm). When ‘pocket closure’ for individual initial PPD thresholds was explored, divided by suprabony/intrabony, suprabony defects consistently exhibited a higher prevalence of ‘pocket closure’ than intrabony defects (Figure 2). This was also confirmed when only cases treated without adjuncts were included (Supporting Information 4).

**TABLE 4:** Periodontal clinical data at baseline and after NSPT according to radiographic defect type.

Defect type	Baseline		After NSPT	
	PPD (mm)	CAL (mm)	PPD reduction (mm)	CAL gain (mm)
	(Mean ± SD)	(Mean ± SD)	(Mean ± SD)	(Mean ± SD)
Suprabony	4.20 ± 2.06	4.75 ± 2.40	0.72 ± 1.79	0.33 ± 1.98
Intrabony	6.13 ± 2.53	6.85 ± 2.84	0.91 ± 2.37	0.31 ± 2.51

### 3.5 Multilevel Analysis

Table 5 shows the results of the multilevel analysis for the outcomes ‘pocket closure’, ‘PPD reduction’ and ‘CAL gain’ while considering data at the site‐ (defect type, initial PPD, therapist, use of adjuncts, number of NSPT visits, instrumentation, re‐evaluation time), tooth level (molar or non‐molar) and patient level (age, gender, diabetes). MINST cases were excluded from the analysis due to low numbers (*n* = 18).

**TABLE 5:** The effect size of each factor with three clinical outcomes (pocket closure, PPD reduction, CAL gain) using multilevel modelling (MLM) adjusted for site‐, tooth‐ and patient‐level clustering effects.

Factor	Clinical outcomes		
	Pocket closure	PPD reduction	CAL gain
	Odds ratio	Beta	Beta
**Site level**			
Defect type, suprabony (intrabony as reference)	2.60 (1.87–3.61)***	0.61 (0.45–0.78)***	0.60 (0.38–0.81)***
Initial PPD	0.51 (0.48–0.55)***	0.52 (0.50–0.55)***	0.47 (0.44–0.51)***
Treatment variables			
Therapist^a^ (postgraduate students as reference)			
*Undergraduate students*	0.79 (0.27–2.28)	0.03 (−0.58–0.63)	0.63 (−0.39–1.65)
*Hygienists or staff members*	1.37 (0.42–4.47)	0.31 (−0.35–0.97)	0.82 (−0.13–1.78)
Use of adjuncts (no adjuncts as reference)	0.99 (0.50–1.97)	0.06 (−0.33–0.45)	−0.21 (−0.69–0.26)
Number of NSPT visits	0.83 (0.63–1.11)	−0.16 (−0.32 to −0.001)*	−0.23 (−0.43 to −0.03)*
Instruments used (both ultrasonic and hand instrumentation as reference)			
*Ultrasonic instrumentation*	1.70 (0.51–5.86)	0.21 (−0.45–0.88)	0.18 (−0.69–1.04)
*Hand instrumentation*	0.99 (0.50–1.97)	−0.05 (−0.44–0.33)	0.21 (−0.28–0.70)
Re‐evaluation time, more than 8 weeks (not more than 8 weeks as reference)	0.72 (0.40–1.31)	−0.31 (−0.66–0.03)	−0.42 (−0.84–0.01)
**Tooth level**			
Tooth type, non‐molar (molar as reference)	1.31 (0.74–2.32)	0.14 (−0.16–0.43)	−0.00 (−0.40–0.40)
**Patient level**			
Age	0.99 (0.97–1.01)	−0.01 (−0.02 to −0.002)*	−0.02 (−0.03 to −0.004)*
Gender, male (female as reference)	0.93 (0.59–1.48)	−0.06 (−0.32–0.20)	−0.06 (−0.39–0.27)
Uncontrolled^b^ diabetes, yes (controlled^c^ diabetes as reference)	1.09 (0.42–2.88)	0.24 (−0.31–0.78)	−0.03 (−0.69–0.64)

*Note*: MLM has further adjusted ‘furcation degree’. Power (PPD reduction): 0.99 (ICC = 0.317); power (CAL gain): 0.99 (ICC0.327); power (pocket closure): 0.981 (ICC = 0.330). The values in bold are statistically significant.

^a^ Postgraduate students (182 patients; 91%); undergraduate students (9 patients; 4.5%); hygienists (3 patients; 1.5%); staff members (6 patients; 3%).

^b^ HbA1c ≥ 6.5%.

^c^ HbA1c < 6.5%.

*p < 0.05, ***p < 0.001.

The results showed that defect type and baseline PPD positively impacted all outcomes at 3 months re‐evaluation (*p* < 0.001). Suprabony defects were 2.60 times more likely to achieve ‘pocket closure’ compared with intrabony defects (95% CI 1.87–3.61, *p* < 0.001).

In addition, suprabony defects had an additional improvement of respectively 0.61 mm (95% CI 0.45–0.78, *p* < 0.001) for PPD reduction and 0.60 mm (95% CI 0.38–0.81, *p* < 0.001) for CAL gain compared with intrabony defects. No treatment‐related variables were statistically significant, except for ‘number of NSPT visits’ on PPD reduction (−0.16, CI −0.32 to −0.001; *p* < 0.05) and CAL gain (−0.23, 95% CI −0.43 to −0.03; *p* < 0.05). Considering patient‐related variables, older age was associated with less PPD reduction (−0.01, 95% CI −0.02 to −0.002) and CAL gain (−0.02, 95% CI −0.3 to −0.004). Similar results were reported for the ‘without adjuncts’ sub‐group, with the addition of a statistically significant effect for ‘re‐evaluation time more than 8 weeks’ (Supporting Information 5).

The estimated relative contribution to the overall variance of ‘pocket closure’ outcome was 64.9% for site‐, 26.1% for patient‐, 9.0% for tooth‐ and 1.0% for treatment‐related factors (Table 5).

Similarly, site‐related factors contributed the most to ‘PPD reduction’ and ‘CAL gain’ outcomes (67.9% and 66.6%, respectively), with decreasing contributions from patient‐, tooth‐ and treatment‐related factors.

When only intrabony defects were included in the analysis, the only factor affecting ‘pocket closure’ outcome was baseline PPD (Supporting Information 6).

## 4 Discussion

At a first glance, this study appears to show that intrabony defects had a higher PPD reduction than suprabony defects after NSPT. This turned out to be entirely due to the initial deeper PPD in intrabony defects. In line with the literature, osseous defects with higher PPD at baseline exhibit a higher PPD reduction and CAL gain at re‐assessment (Lu et al. 2021). When stratified by initial PPD, suprabony defects consistently exhibited a better response compared with intrabony defects, resulting in an increased likelihood (twice as likely) of ‘pocket closure’ for every initial pocket category, irrespective of the use of adjuncts.

This is also confirmed by the present multilevel analysis, which shows that type of defect, as well as the initial PPD, positively impacted all reported clinical outcomes (pocket closure, PPD reduction, CAL gain).

This result clarifies the controversial evidence on the response to NSPT by defect type that emerged in a recent systematic review by our group (Marini et al. 2024). Furthermore, it confirms the reduced response of intrabony defects to standard NSPT, where clinical outcomes are related not only to the initial PPD but also to the complexity of the treatment.

The reason for the poorer response in intrabony defects may lie in the difficulty to disrupt the subgingival biofilm in areas difficult to treat (e.g., furcations, interproximal areas), which are generally associated with deeper pockets (D'Aiuto et al. 2005). This could be particularly pronounced in sites with unfavourable bone morphology (1–2 walls) or with an intrabony component ≥ 3 mm, negatively influencing the healing of deeper bony defects. Interestingly, a recent study by Santamaria et al. (2023) showed a similar molecular profile of GCF between intrabony and suprabony defects, suggesting that the defect morphology, rather than the presence of specific mediators (e.g., IL‐1 α), has the potential to drive the periodontal healing phase.

Tomasi et al. (2007) previously showed that several factors (smoking habit, presence of plaque, multi‐rooted teeth) were negatively associated with PPD reduction after NSPT.

Smoking was associated with a chance of ‘pocket closure’ lower than 30%, while the lower result of NSPT reported for molars was not exclusively due to furcation involvement but was also related to the persistence of plaque in areas with reduced accessibility for subgingival instrumentation (Tomasi et al. 2007).

When the initial PPD was 7–9 mm, Tomasi et al. (2007) reported a reduced chance of ‘pocket closure’ compared to sites with a baseline PPD of 5–6 mm, particularly if the patient was a smoker and the site was located at a molar. This is in line with the present study, where defects with an initial PPD ≥ 7 mm had a lower chance of pocket closure than sites with PPD 4–6 mm. However, the potential interaction with smoking could not be ascertained due to the low prevalence of smokers in the present study (8.5%). Interestingly, in the study of Tomasi et al. (2007) the presence of intrabony defects had no significant effect on ‘pocket closure’, in contrast to the present study.

In addition, sub‐group analysis for intrabony defects showed that baseline PPD was the only factor affecting ‘pocket closure’ outcome and that single‐rooted teeth were 1.88 times more likely to achieve pocket closure than multi‐rooted teeth (when treatment variables were excluded). In line with Kim et al. (2007) NSPT results were more effective in single‐rooted teeth compared to posterior teeth.

The robustness of the data in the present study is supported by comparison with the benchmark represented by the systematic review of Suvan et al. (2020), which showed 57% of ‘pocket closure’ at 3 months (identical to the present study), although average PPD reduction was lower (0.58 mm vs. 1.0 mm in the systematic review).

These results reveal that the NSPT carried out in this Biobank, despite the fact that in the majority of cases the therapists were postgraduate students (and occasionally undergraduate students), is of the standard expected. However, it is worth mentioning that periodontal pockets require a longer healing period after NSPT regardless of defect morphology, reaching 74% of ‘pocket closure’ after 6–8 months (Suvan et al. 2020).

The prevalence of suprabony and intrabony defects in this study is also perfectly in line with the literature, as the number of intrabony defects was around 10% of suprabony defects (Papapanou et al. 1988; Jayakumar et al. 2010).

The present study shows an estimate of relative contribution to the response to NSPT by different sets of factors, similar to what had been reported a couple of decades ago (D'Aiuto et al. 2005).

In the study of D'Aiuto et al. (2005) the main source of variability in PPD reduction following NSPT was attributable to the site‐level parameters (80%), while 12% was at the tooth level and 8% at the patient level.

These data are in line with our multilevel analysis, which demonstrated how the estimated relative contribution to the overall variance of ‘pocket closure’ is mainly related to site‐level factors (64.9%), followed by patient‐level (26.1%) and tooth‐level (9.0%) factors, with a minor contribution of treatment‐related variables (1%).

In line with the literature, instruments used and the treatment approach (full‐mouth or quadrant‐wise) did not affect the efficacy of NSPT (Sanz et al. 2020). In agreement with D'Aiuto et al., these data confirm that the outcomes of NSPT mainly depend on site‐level factors, such as the type of osseous defects. However, this is only an estimate, which is as good as the data included in the analysis and other factors not included in the analysis may yet change the relative contributions.

Regarding patient‐level factors, gender and diabetes did not affect clinical outcomes (including sub‐group analysis for ‘without adjuncts’ and ‘intrabony defects only’), while older age was associated with less PPD reduction and CAL gain, in line with a recent study (Angelov et al. 2024).

A Minimally Invasive Non‐Surgical Therapy (MINST) protocol was recently proposed for the treatment of periodontal pockets. While MINST showed to be effective for intrabony defects, particularly when the osseous defect guarantees clot stability (Nibali et al. 2019; Mehta et al. 2024), a recent split‐mouth study instead showed similar clinical outcomes to traditional NSPT for suprabony defects (Kučič and Gašperšič 2023). However, in this study we excluded MINST cases from the MLM analysis due to a low number. It remains to be elucidated whether the response to MINST of suprabony defects is superior to that of intrabony defects.

The limitations of the present study are due to bias associated with having different operators and skills, as well as several different examiners (who were generally also the operators of each case). This is inherent with point‐of‐care research (Fiore et al. 2011) and has the advantage of allowing generalisability and assessment of the effect of several variables (including operators) on treatment response.

The 3‐month follow‐up is another limitation of the study. However, this re‐evaluation time is due to practical considerations, as in this setting and at the time cases were included, several patients progressed to step 3 periodontal treatment, thus reducing the possibility of observing cases for longer periods after exclusively NSPT.

Furthermore, defect morphology (suprabony/intrabony) was only assessed radiographically and not intra‐surgically. The main strength of this study lies in the novelty of the analyses presented, coupled with the relatively large number of examined sites and the strict radiographic calibration.

The main conclusion that suprabony defects are twice as likely to achieve pocket closure following NSPT and result in 0.6 mm more PPD reduction compared with intrabony defects should be tested in further studies.

## Author Contributions

**Luigi Nibali:** original idea, methodology, critical feedback, writing – original draft, review and editing. **Alessandro Cuozzo:** investigation, radiological analysis, writing – original draft, review and editing. **Mishika Arora:** data curation, data collection; writing – review and editing. **Lorenzo Marini:** data curation, data collection, writing – original draft. **Joshua Hurley:** data curation, data collection, writing – review and editing. **Zainab Malaki:** investigation, data curation, data collection, writing – review and editing. **Jing Kang:** statistical analysis, software, writing – review and editing. **Luca Ramaglia:** critical feedback, writing – review and editing.

## Ethics Statement

The ethics approval with reference number EE/0241 was granted by the East of England–Cambridge East Research Ethics Committee. Specific approval for the release of data and samples was granted by the Biobank Management Committee (Biobank reference 016).

## Conflicts of Interest

The authors declare no conflicts of interest.

## Supporting information


Data S1.

Data S2.

## Data Availability

The data that support the findings of this study are available from the corresponding author (Luigi Nibali) upon reasonable request.
