# Supplementary material for: Differential Response to Non‐Surgical Periodontal Therapy Between Intrabony and Suprabony Defects: A Retrospective Analysis
Source: J Clin Periodontol. 2025 May 19;52(8):1158–66. doi: 10.1111/jcpe.14181 (PMC12259401; doi:10.1111/jcpe.14181)
Supplement: Supplementary file 1 — Data S1. [file JCPE-52-1158-s002.docx]

**SUPPLEMENTAL MATERIAL**

***Supplemental Material 1. Intra-examiner (author AC) comparison***

| **Parameter** | **Intraclass correlation coefficient** | **CI 95%** | |
| --- | --- | --- | --- |
|  |  | **Inf** | **Sup** |
| Root Length | .812 | .410 | .950 |
| Tooth Length | .942 | .786 | .985 |
| Crown Length | .811 | .408 | .949 |
| CEJ – Apical defect | .911 | .684 | .977 |
| Defect Angle | .931 | .747 | .982 |
| Horizontal Component | .996 | .984 | .999 |
| Intrabony Defect | .899 | .646 | .974 |

***Supplemental Material 2. Periodontitis staging and grading***

|  | **Stage 1 (n)** | **Stage 2 (n)** | **Stage 3 (n)** | **Stage 4 (n)** |
| --- | --- | --- | --- | --- |
| Overall (n = 194) | 2; 1% | 7; 3.6% | 103; 53.1% | 82; 42.3% |

|  | **Grade A (n)** | **Grade B (n)** | **Grade C (n)** |
| --- | --- | --- | --- |
| Overall (n = 194) | 4; 2.1% | 18; 9.2% | 172; 88.7% |

|  | **Localized (n)** | **Generalized (n)** |
| --- | --- | --- |
| Overall (n = 194) | 37; 19.1% | 157; 80.9% |

| **Stage** | **Grade** | **Extension** | **n** |
| --- | --- | --- | --- |
| **1** | A | Localized | 1; 0.51% |
|  |  | Generalized | 0; 0 |
|  | B | Localized | 1; 0.51% |
|  |  | Generalized | 0; 0 |
|  | C | Localized | 0; 0 |
|  |  | Generalized | 0; 0 |
| **2** | A | Localized | 0; 0 |
|  |  | Generalized | 1; 0.51% |
|  | B | Localized | 0; 0 |
|  |  | Generalized | 1; 0.51% |
|  | C | Localized | 2; 1.02% |
|  |  | Generalized | 1; 0.51% |
| **3** | A | Localized | 0; 0 |
|  |  | Generalized | 1; 0.51% |
|  | B | Localized | 3; 1.53% |
|  |  | Generalized | 12; 6.12% |
|  | C | Localized | 26; 13.4% |
|  |  | Generalized | 61; 31.44% |
| **4** | A | Localized | 0; 0 |
|  |  | Generalized | 0; 0 |
|  | B | Localized | 0; 0 |
|  |  | Generalized | 0; 0 |
|  | C | Localized | - |
|  |  | Generalized | 82; 41.84% |

***Supplemental Material 3. Instruments and adjuncts (type and dosage) used during NSPT***

| **Instruments (overall patients = 154)** | | | |
| --- | --- | --- | --- |
| Ultrasonic +  hand instrumentation (n) | Hand instrumentation (n) | Ultrasonic instrumentation (n) | MINST (n) |
| 113; 73.4% | 25; 16.2% | 9; 5.8% | 7; 4.5% |
|  | | | |
| **Adjuncts (overall patients = 194)** | | | |
| No (n) | | | 164; 83.7 % |
| Yes (n) | | | 30; 15.3% |
| Azithromycin 500mg OD 3 days (n) | | | 5; 16.7% |
| Azithromycin 250mg OD 3 days (n) | | | 1; 3.3% |
| Amoxicillin 500mg TDS 7 days (n) | | | 3; 10% |
| Amoxicillin 3g 1 hour before NSPT (prophylaxis) (n) | | | 1; 3.3% |
| Amoxicillin 500mg + Metronidazole 400mg TDS 7 days (n) | | | 5; 16.7% |
| Amoxicillin 500mg + Metronidazole 400mg TDS 5 days (n) | | | 9; 30% |
| Amoxicillin 500mg + Metronidazole 400mg TDS 3 days (n) | | | 2; 6.7% |
| Amoxicillin 500mg TDS 7 days + Amoxicillin 500mg TDS 5 days (n) | | | 1; 3.3% |
| Metronidazole 400mg TDS 7 days (n) | | | 2; 6.7% |
| Doxycycline 100mg OD 14 days (n) | | | 1; 3.3% |

**OD**: once daily; **TDS**: three times a day; **NSPT**: Non-Surgical Periodontal Therapy;

**MINST**: Minimally-Invasive Non-Surgical Therapy

***Supplemental Material 4. Proportion of “pocket closure" at re-evaluation time according to initial PPD from 5 to 10 mm divided by suprabony and intrabony defects. All comparisons between suprabony/intrabony are p < 0.001, except 7 mm (p = 0.023), 8 mm (p = 0.064), 9 mm (p = 0.407) and 10 mm (p = 0.038) (including only cases treated with no adjuncts)***

***
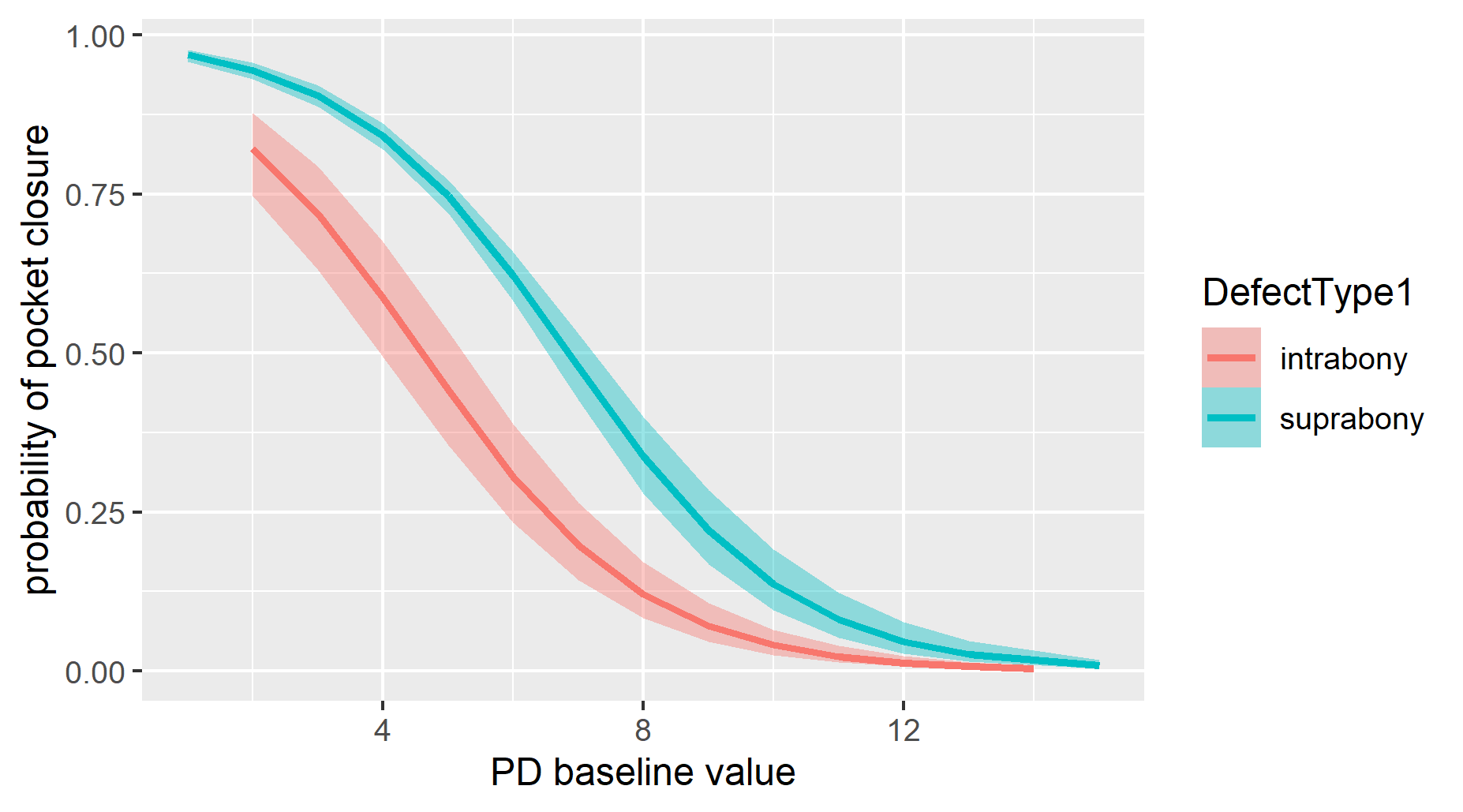
***

***Supplemental Material 5. Subgroup analysis for intrabony defect only. The effect size (odds ratio) of each factor with pocket closure outcome using multilevel modelling (MLM), adjusted for site-, tooth-, and patient-level clustering effect***

| **Factor** | **Pocket closure**  odds ratio  (95% confidence interval) | **Pocket closure with no treatment variables**  odds ratio (95% confidence interval) |
| --- | --- | --- |
| **Site level** | | |
| Initial PPD | **0.62 (0.53-0.72) ***** | **0.57 (0.51-0.65) ***** |
| **Treatment variables** | | |
| Therapist (postgraduate students as reference) |  | |
| Undergraduate students | 3.40 (0.36-31.97) |  |
| Hygienists or staff members | 2.80 (0.33-23.56) |  |
| Use of adjuncts (no adjuncts as reference) | 0.85 (0.31-2.31) |  |
| Number of NSPT visits | 0.70 (0.42-1.17) |  |
| Instruments used (unspecified or both ultrasonic and hand instrumentation as reference) |  | |
| Ultrasonic instrumentation or MINST | 2.62 (0.59-11.67) |  |
| Hand instrumentation | 0.62 (0.16-2.42) |  |
| Re-evaluation time, more than 8 weeks (not more than 8 weeks as reference) | 0.68 (0.27-1.67) |  |
| **Tooth level** | | |
| Tooth type, non-molar (molar as reference) | 0.32 (0.06-1.72) | **1.87 (1.16-3.04) *** |
| **Patient level** | | |
| Age | 1.00 (0.97-1.03) | 1.00 (0.97-1.02) |
| Gender, male (female as reference) | 1.05 (0.51-2.16) | 0.87 (0.45-1.69) |

**Note:** MLM has further adjusted “furcation degree”

*: p < 0.05; ***: p < 0.001
