# Supplementary material for: Differential Response to Non‐Surgical Periodontal Therapy Between Intrabony and Suprabony Defects: A Retrospective Analysis
Source: J Clin Periodontol. 2025 May 19;52(8):1158–66. doi: 10.1111/jcpe.14181 (PMC12259401; doi:10.1111/jcpe.14181)
Supplement: Supplementary file 2 — Data S2. [file JCPE-52-1158-s001.docx]

***Supplemental Material 6. Sub-group analysis for intrabony defect only. The effect size (odds ratio) of each factor with pocket closure outcome using multilevel modelling (MLM), adjusted for site-, tooth-, and patient-level clustering effect***

| **Factor** | **Pocket closure**  odds ratio  (95% confidence interval) | **Pocket closure with no treatment variables**  odds ratio (95% confidence interval) |
| --- | --- | --- |
| **Site level** | | |
| Initial PPD | **0.62 (0.53-0.72) ***** | **0.58 (0.51-0.65) ***** |
| **Treatment variables** | | |
| Therapist^1^ (postgraduate students as reference) |  | |
| Undergraduate students | 3.73 (0.37-37.50) |  |
| Hygienists or staff members | 3.21 (0.35-29.38) |  |
| Use of adjuncts (no adjuncts as reference) | 0.77 (0.26-2.27) |  |
| Number of NSPT visits | 0.69 (0.40-1.17) |  |
| Instruments used (both ultrasonic and hand instrumentation as reference) |  | |
| Ultrasonic instrumentation | 2.75 (0.30-25.57) |  |
| Hand instrumentation | 0.62 (0.15-2.52) |  |
| Re-evaluation time, more than 8 weeks (not more than 8 weeks as reference) | 0.68 (0.27-1.74) |  |
| **Tooth level** | | |
| Tooth type, non-molar (molar as reference) | 0.32 (0.06-1.78) | **1.88 (1.16-3.05)*** |
| **Patient level** | | |
| Age | 1.00 (0.97-1.04) | 1.01 (0.97-1.03) |
| Gender, male (female as reference) | 1.04 (0.48-2.24) | 0.89 (0.45-1.73) |
| Uncontrolled^2^ diabetes, yes (controlled^3^ diabetes as reference) | 1.44 (0.36-5.86) | 1.40 (0.37-5.40) |

**Note:** MLM has further adjusted “furcation degree”

^1^ : postgraduate students (182 patients; 91%); undergraduate students (9 patients; 4.5%); hygienists (3 patients; 1.5%);

staff members (6 patients; 3%)

^2^: HbA1c ≥ 6.5%

^3^: HbA1c < 6.5%

*: p<0.05; ***: p<0.001
